# Autism, Obesity, and PTSD Among Adolescents and Young Adults: An Analysis of National Medicaid Claims Data

**DOI:** 10.1007/s10803-025-06881-1

**Published:** 2025-05-17

**Authors:** Emily Hotez, Rebecca K. Tsevat, Sha Tao, Jenny Mai Phan, Philip Smith, Tammy Shen, Jonas Ventimiglia, Liliana Rivera, Hailey Kissner, Lisa A. Croen, Lindsay Shea

**Affiliations:** 1 University of California, Los Angeles (UCLA), David Geffen School of Medicine, Los Angeles, CA, USA; 2 National Clinician Scholars Program, University of California, Los Angeles, Los Angeles, CA, USA; 3 A.J. Drexel Autism Institute, Drexel University, Philadelphia, PA, USA; 4 Center for Autism, Children’s National Hospital, Washington, DC, USA; 5 Center for Adaptive Systems of Brain-Body Interactions, George Mason University, Fairfax, VA, USA; 6 Division of Research, Kaiser Permanente Northern CA, Pleasanton, CA, USA

**Keywords:** Autism, Health, Obesity, PTSD, Medicaid, Adolescents, Young adults

## Abstract

Autistic individuals disproportionately experience obesity, cardiovascular disease, diabetes, and a range of other adverse health outcomes, relative to both the general population and those with other developmental conditions. These individuals also disproportionately experience Post-Traumatic Stress Disorder (PTSD). Many of these conditions emerge during adolescence and young adulthood (age 15–30). This study analyzed Medicaid claims data (2008–2019) from autistic (*n* = 627,586; *M* age = 17.15 [3.55]) and non-autistic (*n* = 1,223,161; *M* age 19.35 [4.56]) adolescent and young adults. Using logistic regression and adjusting for demographic and clinical characteristics, this study: (1) evaluated associations between the presence of autism, obesity, and other health co-morbidities using the Adolescent and Young Adult (AYA) Hope Comorbidity Index; and (2) tested PTSD as a moderator in these associations. Compared with non-autistic beneficiaries, autistic beneficiaries demonstrated 2.12 (95% CI: 2.09, 2.15) and 2.12 (95% CI: 2.09, 2.16) times the odds of having obesity and other health comorbidities, respectively. PTSD moderated these associations such that autism status was more strongly associated with obesity and health co-morbidities among those *without* a PTSD diagnosis compared to those *with* a PTSD diagnosis. Autistic adolescents and young adults experience higher rates of obesity, health comorbidities, and PTSD relative to their non-autistic counterparts. Future work is needed to explore measurement of stress and trauma beyond PTSD diagnoses and elucidate the precise association between stress and trauma with adverse health outcomes in this population.

Autistic individuals—representing between two and three% of adolescents and adults, respectively, in the U.S. (CDC, 2024; Zablotsky et al., 2019)—experience significant adverse health outcomes across the life course. Multiple studies demonstrate that autistic individuals are disproportionately likely to experience obesity relative to both non-autistic populations and those with other developmental conditions (Byrne et al., 2021; Croen et al., 2015; Flygare Wallén et al., 2018; Phillips et al., 2014; Wallén et al., 2018; Weir et al., 2021). Across the life course, compared with their non-autistic counterparts, autistic individuals have poorer overall health; higher rates of type 2 diabetes, cardiovascular disease, and mortality (Broder-Fingert et al., 2014; Chooi et al., 2019; Croen et al., 2015; Curtin et al., 2014; Davignon et al., 2018; Dhaliwal et al., 2019; Healy et al., 2019; Jones et al., 2016; Kahathuduwa et al., 2019; Matheson & Douglas, 2017; Phillips et al., 2017; Rydzewska et al., 2018; Shedlock et al., 2016; Tint & Weiss, 2018; Tybor et al., 2019; Voulgarakis et al., 2017; Walls et al., 2020); and an overall lower life expectancy (Forsyth et al., 2023; Hirvikoski et al., 2016).

In both autistic and non-autistic populations, health challenges often increase during the developmentally sensitive periods of adolescence and young adulthood (ages 15–30) (Buro et al., 2022; Byrne et al., 2021; Dahl et al., 2018; Fuhrmann et al., 2015). Although sensitive developmental periods have traditionally been conceptualized as occurring in early childhood (Nelson & Gabard-Durnam, 2020), periods of transitions and instability—particularly adolescence and young adulthood—are also pivotal opportunities for intervention (Dahl et al., 2018; Fuhrmann et al., 2015).

These periods, however, are simultaneously characterized by multiple risks and challenges for autistic individuals. The prevalence of obesity during adolescence and young adulthood increases at faster rates for autistic—relative to non-autistic—individuals (Malow et al., 2023), placing autistic individuals at heightened risk for cardiovascular disease, diabetes, and mortality before they enter adulthood. Compounding these challenges, U.S. autistic emerging adults experience a well-documented “services cliff” wherein they can no longer access essential services and supports (e.g., vocational training and mental health counseling) during the transition to adulthood (Ames et al., 2021; Biggs & Carter, 2016; Cheak-Zamora et al., 2013; Feeney et al., 2021; Roux et al., 2023).

Health issues are often linked to social determinants and drivers of health (SDOH), including historical and community factors related to housing, employment status, and food insecurity, as well as persistent and cumulative stressful experiences over the life course. In a recent U.S. analysis of county-level data, SDOH—including education, employment, income, family and social support, and community safety—were the strongest predictors of length and quality of life, exceeding the contributions of health behaviors such as diet and exercise (Explore Health Rankings| Measures & Data Sources, n.d.; Hood et al., 2016). A particularly relevant SDOH indicator for autistic individuals is *chronic stress*—that is, the consequences of persistent and cumulative stressful experiences and trauma over the life course. Indeed, autistic individuals experience pronounced stress due to stigma, discrimination, victimization, trauma, and rejection across the life course (Aubé et al., 2021; Botha & Frost, 2020; Clark et al., 2020; Gillespie-Lynch et al., 2021; McQuaid et al., 2022; Moseley et al., 2021; Pecora et al., 2019; Reuben et al., 2021; Rodriguez et al., 2021).

In non-autistic populations, the association between stress and trauma, obesity, and other health problems is well-established (Cotter & Kelly, 2018; De Vriendt et al., 2009; Razzoli & Bartolomucci, 2016; Siddiqui et al., 2022; Valderhaug & Slavich, 2020; Wiss & Brewerton, 2020). Indeed, stress and trauma lead to obesity via alterations in the hypothalamic–pituitary–adrenal (HPA) axis, which leads to dysregulated cortisol levels that are associated with increased fat deposition, emotional eating, and sedentariness. The cumulative effects of stress- and trauma-related conditions and consequential lifestyle behaviors also contribute to health conditions such as hypertension and cardiovascular disease (Hunger & Tomiyama, 2018) as well as diagnoses of *Post-Traumatic Stress Disorder (PTSD)*. PTSD is broadly defined as intrusive memories, avoidance, negative changes in thinking and mood, and changes in physical and emotional reactions due to experiences of trauma (Hilton et al., 2020; Post-Traumatic Stress Disorder - National Institute of Mental Health (NIMH), n.d.). Numerous studies have established the association of PTSD with a greater risk of coronary heart disease (Edmondson et al., 2013), type 2 diabetes (Miller-Archie et al., 2014), and incident hypertension (Mendlowicz et al., 2023; Rossi & Isnard, 2023).

Research finds that over 40% of autistic individuals report PTSD symptoms within the last month and over 60% report PTSD symptoms at some point in their lifetime; these rates are significantly higher than the 9% of the non-autistic population who experience PTSD following a traumatic event (Rumball et al., 2020). Researchers attribute disproportionate PTSD in autistic populations—in part—to cumulative trauma exposure (Rumball et al., 2020). Indeed, many autistic individuals experience severe social confusion, peer rejection, prevention or punishment of preferred behaviors (e.g., restricted, repetitive interests) and sensory sensitivity to daily stimuli (e.g., lights, loud sounds), which may engender a “traumatic conditioning process” (Haruvi-Lamdan et al., 2020; Rumball et al., 2020; Wood & Gadow, 2010). Autistic children and adolescents also frequently witness or are victims of accidents, disasters, and violence (Mehtar & Mukaddes, 2011). There is preliminary evidence that autistic individuals are more vulnerable to the experiences of stress and trauma (Hoover, 2015; Kildahl et al., 2020), with autistic women and girls experiencing disproportionate vulnerability relative to non-autistic populations and autistic males (Haruvi-Lamdan et al., 2020).

It is currently unknown, however, whether experiences of stress and trauma that lead to PTSD moderate (i.e., strengthen or attenuate) the association between autism status and adverse health outcomes. Additionally, despite preliminary evidence of heightened vulnerability to PTSD in autistic individuals (Hoover, 2015; Kildahl et al., 2020), research is inconclusive as to whether there is a stronger association between PTSD and adverse health outcomes for autistic, relative to non-autistic individuals. Further, there is a lack of research that specifically investigates trauma and health during the sensitive periods of adolescence and young adulthood for autistic individuals.

The current study aimed to understand the role of PTSD in the odds of obesity and other health co-morbidities among autistic adolescent and young adult Medicaid beneficiaries, a population potentially at highest risk for adverse health outcomes. Medicaid is a federal-state partnership that gives states flexibility to structure insurance coverage to meet the specific needs of their populations. Medicaid claims data offers valuable insights into diverse autistic individuals. Medicaid is the largest insurer in the United States for mental health services and is among the only available insurers for many adults with disabilities (About CMS| CMS, n.d.; Scanlon, 2000). Indeed, autistic people are insured at a high rate through Medicaid with over 33% qualifying through low-income in addition to those eligible through waivers (“Brief Report V2,” n.d.). Medicaid enrollees are more racially and ethnically diverse than the overall U.S. population (Race and Ethnicity of the National Medicaid and CHIP Population in 2020, 2020) and are more likely to experience economic hardship relative to those with commercial insurance (Gibbons et al., 2023). As a result, Medicaid claims data analysis and research can elucidate the needs of significantly underserved groups of autistic individuals.

This study tested the following specific aims (depicted in Fig. 1): (1) to compare the odds ratio of the presence of obesity (*primary outcome*) and physical and mental health co-morbidities (*secondary outcome*s) between autistic and non-autistic adolescent and young adult Medicaid beneficiaries; (2) to test the moderating role of PTSD in the association between autism and obesity/health co-morbidities among autistic beneficiaries; and (3) to complete the moderation analysis by determining whether the association between PTSD and the primary and secondary outcomes were stronger for autistic versus non-autistic beneficiaries. We hypothesized that odds of the presence of obesity and health co-morbidities would be higher among autistic individuals (H1) and that the association between autism status and the presence of obesity/health co-morbidities would be significantly stronger for those with PTSD (H2). We hypothesized that the association between PTSD and the presence of obesity/health co-morbidities would be stronger among autistic, versus non-autistic adolescents and young adults (H3).

## Methods

### Overview

This research analyzed administrative claims from the Centers for Medicare & Medicaid Services (CMS). Data were extracted from the 2008–2015 Medicaid Analytic eXtract (MAX) and the 2014–2019 Transformed Medicaid Statistical Information System Analytic Files (TAF). The sample contained Medicaid enrollees from 50 states and Washington, DC, covering both health and mental health services, and including economically disadvantaged individuals and diverse racial and ethnic groups who disproportionately experience poorer healthcare outcomes.

In addition to conducting a secondary data analysis, the first author employed a participatory process for developing the analytic approach. After presenting an initial analytic approach to the team of ten researchers with both lived and professional autism and Medicaid experience—as well as those with specific Medicaid data analysis expertise—the first author held a series of collaborative virtual meetings with all co-authors to refine and enhance the analytic approach and interpretation of findings. The authors met for six one-hour sessions and conducted additional virtual collaborations asynchronously. This process enhanced the extent to which the analytic plan reflected the needs, experiences, and priorities of autistic individuals.

### Sample

The study sample characteristics are described in Table 1. The sample featured autistic Medicaid adolescent and young adult (age 15–30) Medicaid beneficiaries (*n* = 627,586) and a non-autistic random sample of beneficiaries without intellectual disability (*n* = 1,223,161). To be eligible for overall sample inclusion, individuals had to be 15 to 30 years old at any point within the study period (2008–2019) and have at least twelve months of observed Medicaid enrollment in the age range during the study period. Relative to the non-autistic sample, the autistic sample was slightly younger (Autistic: *M* age = 17.15 [3.55]; Non-Autistic: *M* age = 19.35 [4.56]); disproportionately Male (Autistic: Male = 75.7%; Non-Autistic: Male = 36.40%); and disproportionately White (Autistic: White = 56.04%; Non-Autistic: White = 39.49%).

### Measures

All variables are described below and defined in Table 2.

### Predictor: Autism

To be categorized as autistic, individuals had to have at least one inpatient, or two other claims, associated with an autism diagnosis code (ICD-9 299.xx, ICD-10 F84.x) in the study period, in alignment with the algorithm defined in the Chronic Conditions Data Warehouse (CCW) and the methodology utilized in autism claims-based research (Grosse et al., 2021). Multiple studies have confirmed the validity of an autism diagnosis observed in claims data (Burke et al., 2014; Coo et al., 2012; Dodds et al., 2009; Straub et al., 2021). The positive predictive value of an autism diagnosis within healthcare claims in the community is very high (97-98%) (Burke et al., 2014).

### Primary Outcome: Obesity

To be categorized as having obesity, individuals had to have one inpatient or two other claims associated with a diagnosis code corresponding to obesity during the study period (Table 2). These codes are consistent with validated algorithms and previous research on obesity (Dashputre et al., 2020; Lloyd et al., 2015).

### Secondary Outcome: Health Co-Morbidities

The Adolescent & Young Adult Health Outcomes & Patient Experience Study (AYA HOPE) created the AYA HOPE comorbidity index. This index is an aggregate variable based on 14 categories of health conditions (i.e., cardiovascular, hypertension, asthma/respiratory, endocrine, diabetes mellitus, liver, gastrointestinal, hematologic, HIV/AIDS, mental health [excluding PTSD], neurologic, obesity/overweight, renal, and rheumatologic/autoimmune) relevant for young adults (Wu et al., 2015). Although primarily featured in cancer research, in the current study, the AYA HOPE categories were operationally defined and created based on these conditions in the claims data (Table 2). Enrollees who met the sub-condition requirements for any of the listed conditions were considered to have that condition. The AYA HOPE comorbidity index was treated as a binary variable: individuals with 0 to 3 conditions were categorized as having “less severe conditions,” while those with 4 or more conditions were categorized as having “more severe conditions.”

### Moderator: PTSD

To be categorized as having PTSD, individuals had to have at least one in-patient, or two other claims associated with an PTSD diagnosis code (ICD-9 309.81 ICD-10 F43.10, F43.11, F43.12) within the study period. These codes are consistent with validated algorithms and previous research on PTSD (Gravely et al., 2011).

### Covariates

We controlled for age, race, ethnicity, and sex-based on the group differences observed between autistic and non-autistic beneficiaries (see SM3). Due to their associations with experiences of structural inequities, racism, and environmental injustice, race and ethnicity were utilized as control variables to account for potential differences in these experiences. Indeed, there are documented racial and ethnic disparities in autism diagnosis, obesity occurrence, and stress (Aylward et al., 2021; Magaña et al., 2016; Maye et al., 2022; Schott et al., 2022; Weitlauf et al., 2023). We also adjusted for Medicaid eligibility group, enrolled month, and state.

### Analytic Approach

Aim 1 was to compare autistic adolescent and young adult Medicaid beneficiaries versus their non-autistic counterparts on obesity and health co-morbidities in the project period. We tested this aim by conducting logistic regression, comparing autistic versus non-autistic Medicaid beneficiaries on their odds of obesity (*primary outcome*), adjusting for age group, sex, race and ethnicity, Medicaid eligibility group, enrolled month, and state. Logistic regression was also used to compare individuals with and without autism on odds of AYA HOPE comorbidities (4 + vs. 0–3 [*secondary outcome*]), adjusting for the same covariates. Aim 2 aimed to test the moderating role of PTSD in the association between autism and the presence of obesity and between autism and the presence of AYA HOPE comorbidities, again adjusting for the same covariates. Aim 3 featured analyses to determine whether the association between PTSD and the primary and secondary outcomes were stronger for autistic versus non-autistic beneficiaries. Both adjusted and unadjusted models were formulated. Additional analyses included checks to identify whether trends upheld for both males and females.

## Results

Overall, 13.2% of autistic Medicaid enrolled beneficiaries had an obesity diagnosis, relative to 6.8% in the comparison group. With respect to the HOPE co-morbidities, 14.74% and 5.12% of autistic and non-autistic beneficiaries had four or more HOPE co-morbidities, respectively. As depicted in SM3–5, findings were consistent when disaggregated by sex.

As depicted in the Supplemental Material (SM1–2), the most prevalent AYA co-morbidities experienced by autistic individuals were mental health issues (excluding PTSD, 64.75%); neurologic conditions (39.23%); and cardiovascular conditions (17.10%) and the most prevalent AYA co-morbidities in the non-autistic individuals were mental health issues (excluding PTSD, 22.65%); neurologic conditions (17.06%); and gastrointestinal issues (8.77%). As indicated in Table 1, the PTSD prevalence for autistic versus non-autistic beneficiaries was 6.37% vs. 2.23%.

### Aim 1

Findings are presented in Table 3. Compared with non-autistic Medicaid beneficiaries, autistic Medicaid beneficiaries had 2.12 (95% CI: 2.09–2.15) times the odds of having a diagnosis of obesity and 2.12 (95% CI: 2.09–2.16) times the odds of having four or more HOPE comorbidities (Table 3). Females exhibited higher rates of all conditions assessed (PTSD, obesity, and severe HOPE conditions) (SM3–5).

### Aims 2 and 3

Findings are presented in Table 4.

### Primary Outcome: Obesity

A likelihood ratio test for the interaction between PTSD and autism status in association with obesity diagnosis was statistically significant (*p* = 0.0002). PTSD moderated the association between autism and obesity: autism was slightly more strongly associated with obesity among those *without* PTSD compared to those *with* PTSD. Specifically, among those without PTSD, autism status was associated with 2.06 times greater odds of an obesity diagnosis (95% CI: 2.03, 2.09); among those with PTSD, autism status was associated with 1.80 times greater odds of an obesity diagnosis (95% CI: 1.72, 1.89). Among autistic enrollees, PTSD (vs. no PTSD) was associated with 2.20 times greater odds of an obesity diagnosis (95% CI: 2.14, 2.25). Approximately the same finding was detected for non-autistic enrollees (OR = 2.18; 95% CI: 2.11, 2.25). Trends remained consistent between males and females.

### Secondary Outcome: Health Co-Morbidities

The likelihood ratio test for the interaction between autism status and PTSD was statistically significant (*p* < 0.0001). PTSD moderated the association between autism status and HOPE co-morbidity scores: there was a stronger association between autism status and HOPE co-morbidities among those *without* PTSD compared to those *with* PTSD. Specifically, among those without PTSD, autism was associated with 2.02 times greater odds of a 4 + HOPE co-morbidity score (95% CI: 2.00, 2.06); among those with PTSD, autism status was associated with 1.49 times greater odds of a 4 + HOPE co-morbidity score (95% CI: 1.43, 1.56). Among autistic enrollees, PTSD was associated with 3.58 times greater odds of a 4 + HOPE co-morbidity score (95% CI: 3.49, 3.66). Among non-autistic enrollees, PTSD was associated with 5.03 times greater odds of a 4 + HOPE comorbidity score (95% CI: 4.88, 5.19). Trends remained consistent between males and females.

## Discussion

The current study featured a secondary data analysis of Medicaid claims data from 2008 to 2019 to test the association between autism status, PTSD, obesity, and health co-morbidities among adolescent and young adult Medicaid beneficiaries. This study found that autistic—compared to non-autistic—adolescents and young adults were disproportionately likely to have a diagnosis of obesity, four or more health co-morbidities, and PTSD. Furthermore, autism status was slightly more strongly associated with obesity and health co-morbidities among those *without* a PTSD diagnosis. Our findings align with previous research demonstrating that autistic individuals are disproportionately likely to experience obesity and other adverse health outcomes, particularly during key developmental transitions such as adolescence and young adulthood (Buro et al., 2022; Croen et al., 2015). Compared to prior studies, this study is novel in that it featured a community sample with a much larger sample size and focused on a population that may experience disproportionately negative SDOH.

The moderation findings from Aims 2 and 3 detected in this study require further research as they were inconsistent with our hypothesis. There are several possible interpretations. Many autistic individuals *without* a PTSD diagnosis may actually meet criteria for PTSD but have not received an official diagnosis, potentially due to stronger prioritization of the primary diagnosis of autism. This group may be a particularly underserved subgroup and warrant additional attention in the research. Indeed, despite the high rates of trauma and PTSD in autistic individuals—particularly during adolescence and young adulthood—PTSD often goes unrecognized (Reuben et al., 2022). Patients likely to have undiagnosed PTSD are also observed to have increased rates of PTSD complications, including sleep disturbances, suicidal thoughts, and substance use. Machine learning research suggests that PTSD symptoms may be exacerbated among those without a formal PTSD diagnosis, potentially due to management being directed primarily toward addressing individual symptoms (Gagnon-Sanschagrin et al., 2022). Qualitative studies find that autistic individuals with PTSD are less connected to services and treatment providers (Kildahl et al., 2020). Many of the traumatic life events autistic individuals experience (e.g., negative experiences in diagnostic and therapeutic contexts) may not typically be considered traumatic events for the non-autistic population. These findings from previous research provide support that autistic people may not receive the help they need for likely PTSD (Rumball et al., 2020). Thus, although the informative presence bias might suggest that autistic individuals are more likely to obtain a PTSD diagnosis, given their increased interactions with the healthcare system (McGee et al., 2022), it is possible that the opposite trend is occurring here because of challenges with recognition, diagnosis, and treatment in this population.

Alternatively, many autistic individuals may be accustomed to trauma and even if they have consistent access to high-quality healthcare and may be unaware of the potential to address or prevent these circumstances through receiving a stress- or trauma-related diagnosis. Indeed, individuals with intellectual and/or developmental disabilities such as autism experience stigma (and othering) beginning in childhood, initially in the form of *implicit stigma*: insidious—rather than direct or overt—negative labeling that can result in strained or uncomfortable social interactions, lower quality of life and self-esteem, and other consequences (Aubé et al., 2021; Blundell et al., 2016; Ditchman et al., 2016; Link & Phelan, 2006). This stigma may be interwoven throughout all aspects of autistic individuals’ lives, making it difficult to disentangle stigma-related stress and/or trauma that can potentially be ameliorated. To address these inconclusive findings, future research should test multiple indicators of stress and trauma in relation to health outcomes, as PTSD may be a useful yet incomplete index of these experiences.

Our analyses revealed that autistic and non-autistic individuals with PTSD were approximately equally likely to experience obesity. This finding aligns with research conducted with the general population that links stress and trauma—which may accumulate and contribute to a verified PTSD diagnosis—to obesity (Edmondson et al., 2013; Hunger & Tomiyama, 2018). This is one of the first studies, however, that documents an equivalent relationship between PTSD and health outcomes in autistic adolescents and young adults. Given that 60% of autistic individuals report PTSD at some point in their lifetime (Rumball et al., 2020) and autistic individuals may be particularly vulnerable to stress (Haruvi-Lamdan et al., 2020), our research suggests that experiences of stress and trauma may be important—and largely untapped—upstream obesity intervention targets for autistic populations.

Additionally, the association between PTSD and co-morbidities was stronger for non-autistic—relative to autistic—adolescents and young adults. Again, this finding was inconsistent with our hypothesis, and there are multiple potential interpretations of this finding. It may be the case that there are myriad circumstances in autistic individuals’ lives contributing to their health, and stress is simply one of many. As a result, PTSD may have a weaker effect on specific health outcomes for autistic—relative to non-autistic— individuals. Indeed, research has cited multiple reasons for obesity and other health outcomes in autistic individuals, including medication usage, food sensitivities, barriers to physical activity, and co-occurring genetic conditions (Buro et al., 2022; Curtin et al., 2014). The current study found that the most prevalent AYA co-morbidities experienced by autistic individuals were mental health issues (excluding PTSD), neurologic conditions, and cardiovascular conditions, which may interact and compound. Future research should explore these factors and identify the most impactful predictors of obesity and adverse health outcomes.

Although not explored in this research, the association between PTSD and adverse health outcomes in autistic patients may be uniquely linked to the concept of *minority stress*– that is, experiences of stigma-related stress due to decreased social standing. Over time, minority stress leads to increased allostatic load and significant adverse health outcomes (Botha & Frost, 2020; Parra & Hastings, 2018; Seeman et al., 1997). Autistic individuals with multiple marginalized intersectional identities (e.g., autistic racial and ethnic minorities and/or LGBTQ + individuals) often experience heightened minority stress. In effect, they experience even greater adverse health outcomes and unmet healthcare needs (Botha & Gillespie-Lynch, 2022; Cohen et al., 2022; Hotez et al., 2024; Mulcahy et al., 2022). Given that our sample demonstrated a high degree of sociodemographic diversity, it is possible that many individuals in our sample experience a high level of minority stress, due to identification with multiple marginalized statuses. Research supports that certain autistic subgroups (e.g., autistic females) may be particularly vulnerable to stress (Haruvi-Lamdan et al., 2020). Our own analyses revealed that females exhibited higher rates of all conditions assessed (PTSD, obesity, and severe HOPE conditions). Given findings in previous research—as well as in the current study— there is a need for future research to investigate the unique experiences of autistic individuals who have multiple marginalized identities.

This study had several strengths that offered unique contributions to the field. The use of Medicaid claims data allowed for the examination of a large and heterogenous population of autistic individuals. The sample had sociodemographic and health characteristics similar to those of Medicaid enrollees overall, contributing to the overall generalizability of the results to individuals with Medicaid insurance coverage. Moreover, the use of Medicaid claims data in this study has important policy implications. As state Medicaid agencies increasingly integrate coverage for SDOH-related interventions into value-based payment models, (Spencer et al., n.d.), there is a growing need to define the role of SDOH in physical health outcomes. As demonstrated in our study, this need is particularly pronounced for autistic individuals, who experience disproportionately high rates of SDOH-related stressors and adverse health outcomes. In addition to these limitations, previous research has identified that PTSD is independently associated with both a higher risk of weight gain and loss (LeardMann et al., 2015). It may be beneficial for future research to disentangle these linkages for autistic individuals.

Finally, this is the first study to apply the AYA HOPE co-morbidity index to a sample of autistic individuals, demonstrating a strategy for examining holistic health in this population. The use of this index allowed our study to test an aggregate variable based on 14 categories of health conditions relevant for young adults (Wu et al., 2015). Continued utilization of this aggregate measure may have ramifications for future research examining holistic mental and physical health outcomes in this population.

### Limitations

This study also had several limitations. This analysis was cross-sectional, and as such, it was not possible to ascertain a causal relationship between the variables tested as part of this analysis. Nevertheless, the moderating pathway is a potentially useful way to study how the health outcomes of autistic individuals may be differentially affected by the experience of PTSD. Additionally, the use of Medicaid claims data is limited in its ability to provide information about contexts surrounding the diagnoses. It is unclear, for example, whether PTSD diagnoses were related to autism or to other unrelated traumatic events. Furthermore, PTSD in general is underdiagnosed (Meltzer et al., 2012), indicating that our analysis may under-report actual PTSD cases. Indeed, lack of time and patient financial burden have been identified as the strongest barriers to diagnosis and treatment of PTSD among primary care physicians (Meltzer et al., 2012). Given this finding—and the reliance on a Medicaid sample that experiences significant patient financial burden— the use of PTSD as a marker for chronic stress may underestimate the true prevalence and effects of chronic stress for autistic individuals.

## Conclusion

This study is an important contribution to the literature because it elucidates the need to further investigate additional markers of stress among particularly underserved groups of autistic individuals during critical developmental windows. Specifically, this study demonstrates that autistic adolescents and young adults have significantly higher rates of obesity and other chronic health conditions than their non-autistic peers. Additionally, PTSD moderates the associations between autism and these adverse health outcomes, with stronger associations between autism status and adverse health outcomes for those without a PTSD diagnosis. As there may be diagnostic barriers to identifying trauma specific to this population (Reuben et al., 2022), multidimensional, individualized assessment strategies may be necessary to recognize PTSD or trauma-related symptoms (Kildahl et al., 2020). Future work is needed to develop sensitive self-report measures and validate existing measures for assessing trauma in autistic adolescents and young adults (Hoover, 2015). Additionally, future efforts should be directed toward developing interventions that can target experiences of stress and trauma to mitigate health disparities and improve health trajectories for this population.

## Supplementary Material

Supplementary Material 1

Supplementary Material 2

Supplementary Material 3

Supplementary Material 4

Supplementary Material 5

The online version contains supplementary material available at https://doi.org.10.1007/s10803-025-06881-1.

## Figures and Tables

**Fig. 1:**
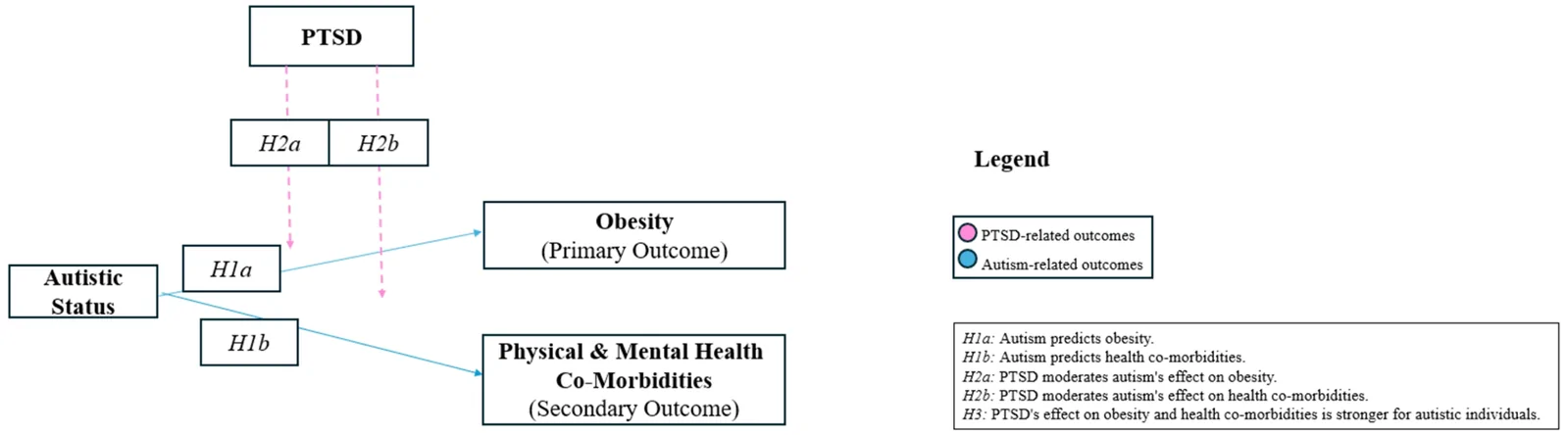
Conceptual model

**Table 1:** Demographic for medicaid enrollees (age 15–30) comparing autistic versus non-autistic beneficiaries (2008–2019)

	Autistic		Non-Autistic	
	*N* = 627,586		*N* = 1,223,161	
	*N*	%	*N*	%
**Age (mean/SD)**	17.15	3.55	19.35	4.56
**Age Group**				
15–19	505,357	80.52	721,977	59.03
20–24	77,057	12.28	269,801	22.06
25–30	45,172	7.20	231,383	18.92
**Sex**				
Male	474,940	75.68	445,278	36.40
Female	152,646	24.32	777,883	63.60
**Race and Ethnicity**				
White	351,686	56.04	485,500	39.69
Black	94,403	15.04	277,200	22.66
Asian/Pacific Islander	16,940	2.70	51,366	4.20
Hispanic/Latino	79,617	12.69	330,276	27.00
Multi-race	5,380	0.86	4,858	0.40
Native Indian/Alaska Native	5,870	0.94	17,747	1.45
Missing*	73,690	11.74	56,214	4.60
**Eligibility Group**				
Poverty	101,974	16.25	724,102	59.20
Disability	435,997	69.47	118,291	9.67
Other	89,615	14.28	380,768	31.13
**Total enrolled months (mean/SD)**	61.10	36.77	38.72	25.06
**Total enrolled months**				
12	8,541	1.36	50,624	4.14
13–36	208,454	33.22	672,456	54.98
37+	410,591	65.42	500,081	40.88
**PTSD**	39,968	6.37	27,272	2.23
**Obesity**	82,906	13.21	83,711	6.84
**HOPE (mean/SD)**	1.87	1.67	0.85	1.30
**HOPE (binary)**				
Less Severe	535,095	85.26	1,160,582	94.88
More Severe	92491	14.74	62579	5.12

* Note: The only variable with significant missing data was race/ethnicity. Missing values were treated as a separate category in subsequent models presented in the current paper

**Table 2:** Key variables and operational definitions

Variables	Example ICD codes
**Primary Outcome**: Obesity-Related Diagnoses	• Obesity unspecified (E66.9) • BMI-related diagnoses (V85.3– V85.45; Z68.3– Z68.45) • Overweight and obesity (278.0–278.03)
**Secondary Outcome**: HOPE	• *See* Supplemental Material
**Primary Predictor**: Autism	• Autistic disorder (299.0–299.9; F84.0 - F84.9)
**Moderator Variable**: PTSD	• PTSD (ICD-9 309.81 ICD-10 F43.10, F43.11, F43.12)
**Controls**	
**Demographic**	• Race/ethnicity, gender, age, insurance type/eligibility
Clinical	• Support needs (including medications), Language abilities, Cognitive development

**Table 3:** Prevalence and odds of obesity and HOPE more severe condition among medicaid enrollees (age 15–30) among autistic and non-autistic beneficiaries (2008–2019)

	Autistic								Non-Autistic	
	
	*N* = 627,586								*n* = 1,223,161	
	
	N	%	uOR^1^	95% CI		aOR^2^	95% CI		N	%
Obesity	82,906	13.21	2.07	2.05	2.09	2.12	2.09	2.15	83,711	6.84
HOPE^3^	92,491	14.74	3.21	3.17	3.24	2.12	2.09	2.16	62,579	5.12

1 Unadjusted OR comparing autistic versus non-autistic beneficiaries

2 Logistic regression adjusted for age group, sex, race/ethnicity, Medicaid eligibility group, enrolled month group, and state

3 Odds comparing more severe condition to less severe condition

**Table 4:** Prevalence and odds of obesity and HOPE index by PTSD among medicaid enrollees (age 15–30) comparing autistic versus non-autistic beneficiaries (2008–2019)

		Autistic					Non-Autistic					Comparison between autistic versus non-autistic			Moderation
		*N* = 627,586					*N* = 1,223,161								
				
				Within Group					Within Group						
			
		N	%	aOR^1^	95% CI		N	%	aOR^1^	95% CI		aOR^2^	95% CI		p-value^3^
Obesity	With PTSD	9,707	24.29	2.196	2.14	2.253	4,695	17.22	2.180	2.10	2.25	1.803	1.71	1.892	0.0002
	No PTSD	73,199	12.46	Ref			79,016	6.61	Ref			2.061	2.02	2.094	
HOPE	With PTSD	13,590	34.00	3.575	3.49	3.662	7,327	26.87	5.028	4.87	5.18	1.491	1.42	1.558	< 0.0001
	No PTSD	78,901	13.43	Ref			55,252	4.62	Ref			2.022	1.98	2.057	

1 Logistic regression comparing the odds of Obesity / more severe condition of HOPE among those with PTSD to those without PTSD, within the autistic/non-autistic samples, adjusting for age group, sex, race/ethnicity, Medicaid eligibility group, enrolled month group, and state

2 Logistic regression comparing the odds of Obesity / more severe condition of HOPE among autistic versus non-autistic samples, within the PTSD/no PTSD smaple, adjusting for age group, sex, race/ethnicity, Medicaid eligibility group, enrolled month group, and state

3 The Likelihood Ratio Test (LRT) for the interaction term of AUTISM*PTSD (Significant)
