# Supplementary material for: Autism, Obesity, and PTSD Among Adolescents and Young Adults: An Analysis of National Medicaid Claims Data
Source: J Autism Dev Disord. 2025 May 17;56(11):4392–403. doi: 10.1007/s10803-025-06881-1 (PMC12434421; doi:10.1007/s10803-025-06881-1)
Supplement: Supplementary file 1 [file 10803_2025_6881_MOESM1_ESM.docx]

**SM1: Table appendix. HOPE 14 categories among Medicaid enrollees (age 15-30) comparing autistic versus non-autistic beneficiaries (2008-2019)**

|  | **Autistic** | | **Non-Autistic** | |
| --- | --- | --- | --- | --- |
|  | N = 627,586 | | N = 1,223,161 | |
|  | N | % | N | % |
| **HOPE (14 level)** |  |  |  |  |
| 0 | 117,891 | 18.78 | 690,641 | 56.46 |
| 1 | 201,161 | 32.05 | 267,649 | 21.88 |
| 2 | 137,807 | 21.96 | 134,764 | 11.02 |
| 3 | 78,236 | 12.47 | 67,528 | 5.52 |
| 4 | 43,475 | 6.93 | 33,039 | 2.70 |
| 5 | 24,112 | 3.84 | 16,232 | 1.33 |
| 6 | 12,871 | 2.05 | 7,433 | 0.61 |
| 7 | 6,636 | 1.06 | 3,434 | 0.28 |
| 8 | 3,233 | 0.52 | 1,529 | 0.13 |
| 9 | 1,469 | 0.23 | 631 | 0.05 |
| 10 | 479 | 0.08 | 206 | 0.02 |
| 11 | 159 | 0.03 | *censor for other cells* | |
| 12 | 38 | 0.01 | * | * |
| 13 | * | * | 0 | 0.00 |
| 14 | * | * | * | * |
| **HOPE 14 Categories** |  |  |  |  |
| Cardiovascular | 107,328 | 17.10 | 86,034 | 7.03 |
| Diabetes Mellitus | 32,220 | 5.13 | 26,188 | 2.14 |
| Endocrine | 46,971 | 7.48 | 41,135 | 3.36 |
| Gastrointestinal | 71,862 | 11.45 | 107,261 | 8.77 |
| HIV/AIDS | 1,448 | 0.23 | 4,216 | 0.34 |
| Hypertension | 50,458 | 8.04 | 45,837 | 3.75 |
| Liver | 16,666 | 2.66 | 17,995 | 1.47 |
| Mental Health | 406,337 | 64.75 | 277,017 | 22.65 |
| Neurologic | 246,221 | 39.23 | 208,718 | 17.06 |
| Obesity/Overweight | 82,906 | 13.21 | 83,711 | 6.84 |
| Renal | 25,073 | 4.00 | 21,312 | 1.74 |
| Asthma/Respiratory | 82,841 | 13.20 | 115,571 | 9.45 |
| Rheumatologic/Autoimmune | 3,607 | 0.57 | 4,506 | 0.37 |
| hematologic | 2,019 | 0.32 | 3,018 | 0.25 |
