# Supplementary material for: Autism, Obesity, and PTSD Among Adolescents and Young Adults: An Analysis of National Medicaid Claims Data
Source: J Autism Dev Disord. 2025 May 17;56(11):4392–403. doi: 10.1007/s10803-025-06881-1 (PMC12434421; doi:10.1007/s10803-025-06881-1)
Supplement: Supplementary file 5 [file 10803_2025_6881_MOESM5_ESM.docx]

**SM5. Prevalence and odds of Obesity and HOPE Index by PTSD among Medicaid enrollees (age 15-30) comparing autistic versus non-autistic beneficiaries (2008-2019) by sex**

|  |  |  | **Autistic** | | | | | **Non-Autistic** | | | | | **Comparison between autistic versus non-autistic** | | | **Moderation** |
| --- | --- | --- | --- | --- | --- | --- | --- | --- | --- | --- | --- | --- | --- | --- | --- | --- |
|  |  |  |  |  | **Within Group** | | |  |  | **Within Group** | | |  |  |  |  |
|  |  |  | **N** | **%** | **aOR^1^** | **95% CI** | | **N** | **%** | **aOR^1^** | **95% CI** | | **aOR^2^** | **95% CI** | | **p-value^3^** |
| **Obesity** | **Male** | **With PTSD** | 4,683 | 19.43* | 1.912 | 1.847 | 1.979 | 571 | 8.51 | 1.994 | 1.823 | 2.180 | 2.304 | 2.086 | 2.543 | 0.4542 |
|  |  | **No PTSD** | 51,806 | 11.49 | Ref | | | 16,947 | 3.86 | Ref | | | 2.643 | 2.584 | 2.704 |  |
|  | **Female** | **With PTSD** | 5,024 | 31.66 | 2.599 | 2.500 | 2.701 | 4,124 | 20.05 | 2.190 | 2.112 | 2.272 | 1.676 | 1.582 | 1.774 | <.0001 |
|  |  | **No PTSD** | 21,393 | 15.64 | Ref | | | 62,069 | 8.20 | Ref | | | 1.645 | 1.606 | 1.684 |  |
| **HOPE** | **Male** | **With PTSD** | 7,038 | 29.20 | 3.289 | 3.187 | 3.394 | 1,242 | 18.52 | 5.279 | 4.926 | 5.656 | 1.556 | 1.441 | 1.680 | <.0001 |
|  |  | **No PTSD** | 55,757 | 12.37 | Ref | | | 13,603 | 3.10 | Ref | | | 2.357 | 2.302 | 2.414 |  |
|  | **Female** | **With PTSD** | 6,552 | 41.29 | 3.947 | 3.800 | 4.099 | 6,085 | 29.59 | 4.908 | 4.741 | 5.080 | 1.469 | 1.392 | 1.550 | <.0001 |
|  |  | **No PTSD** | 23,144 | 16.92 | Ref | | | 41,649 | 5.50 | Ref | | | 1.713 | 1.670 | 1.758 |  |

^1^ Logistic regression comparing the odds of Obesity / more severe condition of HOPE among those with PTSD to those without PTSD, within the autistic/non-autistic samples, adjusting for age group, sex, race/ethnicity, Medicaid eligibility group, enrolled month group, and state.

^2^ Logistic regression comparing the odds of Obesity / more severe condition of HOPE among autistic versus non-autistic samples, within the PTSD/no PTSD sample, adjusting for age group, sex, race/ethnicity, Medicaid eligibility group, enrolled month group, and state.

^3^ The Likelihood Ratio Test (LRT) for the interaction term of AUTISM*PTSD (Significant)

* Among autistic beneficiaries, 19.43% of those with PTSD diagnosis had an obesity diagnosis
