# Supplementary material for: Autism, Obesity, and PTSD Among Adolescents and Young Adults: An Analysis of National Medicaid Claims Data
Source: J Autism Dev Disord. Author manuscript; Available in PMC 2025 Sep 15. (PMC12434421; doi:10.1007/s10803-025-06881-1)
Supplement: Supplementary Material 2 [file NIHMS2101657-supplement-Supplementary_Material_2.docx]

**SM2. AYA HOPE ICD Codes**

| **Co-morbidity** | **ICD-9** | **Matching ICD-10** | **ICD-9 from CCW** | **ICD-10 Description** | **Reference** | **CCW Comorbidity Name** | |  |
| --- | --- | --- | --- | --- | --- | --- | --- | --- |
| **Cardiovascular** |  |  |  |  |  |  |  |  |
| *Ischemic Heart* |  |  | 410.00, 410.01, 410.02, 410.10, 410.11, 410.12, 410.20, 410.21, 410.22, 410.30, 410.31, 410.32, 410.40, 410.41, 410.42, 410.50, 410.51, 410.52, 410.60, 410.61, 410.62, 410.70, 410.71, 410.72, 410.80, 410.81, 410.82, 410.90, 410.91, 410.92, 411.0, 411.1, 411.81, 411.89, 412, 413.0, 413.1, 413.9, 414.00, 414.01, 414.02, 414.03, 414.04, 414.05, 414.06, 414.07, 414.12, 414.2, 414.3, 414.4, 414.8, 414.9 | I20.0, I20.1, I20.8, I20.9, I21.01, I21.02, I21.09, I21.11, I21.19, I21.21, I21.29, I21.3, I21.4, I21.A1, I21.A9, I22.0, I22.1, I22.2, I22.8, I22.9, I23.0, I23.1, I23.2, I23.3, I23.4, I23.5, I23.6, I23.7, I23.8, I24.0, I24.1, I24.8, I24.9, I25.10, I25.110, I25.111, I25.118, I25.119, I25.2, I25.3, I25.41, I25.42, I25.5, I25.6, I25.700, I25.701, I25.708, I25.709, I25.710, I25.711, I25.718, I25.719, I25.720, I25.721, I25.728, I25.729, I25.730, I25.731, I25.738, I25.739, I25.750, I25.751, I25.758, I25.759, I25.760, I25.761, I25.768, I25.769, I25.790, I25.791, I25.798, I25.799, I25.810, I25.811, I25.812, I25.82, I25.83, I25.84, I25.89, I25.9 | CCW | Ischemic Heart Disease |  |  |
| *Disease of Pericardium* | 423.9 | I31.9 |  |  | ^1^ |  |  |  |
| *Valve Disorder* | 424.0 | I34.0, I34.8 |  |  | ^1^ |  |  |  |
| *Arrhythmias* | 785.0, 785.2, 427.32, 427.69 | R00.0, R01.1, I48.92, I49.3, I49.49 |  |  | ^1^ |  |  |  |
| *Congestive Heart Failure* |  |  | 398.91, 402.01, 402.11, 402.91, 404.01, 404.03, 404.11, 404.13, 404.91, 404.93, 428.0, 428.1, 428.20, 428.21, 428.22, 428.23, 428.30, 428.31, 428.32, 428.33, 428.40, 428.41, 428.42, 428.43, 428.9 | I09.81, I11.0, I13.0, I13.2, I50.1, I50.20, I50.21, I50.22, I50.23, I50.30, I50.31, I50.32, I50.33, I50.40, I50.41, I50.42, I50.43, I50.810, I50.811, I50.812, I50.813, I50.814, I50.82, I50.83, I50.84, I50.89, I50.9 | CCW | Heart Failure |  |  |
| *Blood Clots* | 453.2 | I82.220, I82.221 |  |  | ^1^ |  |  |  |
| *Peripheral Vascular Disease* | - | - |  |  | ^1^ |  |  |  |
| *Lipid Disorder* |  |  | 272.0, 272.1, 272.2, 272.3, 272.4 | E78.0, E78.00, E78.01, E78.1, E78.2, E78.3, E78.4, E78.41, E78.49, E78.5 | CCW | Hyperlipidemia |  |  |
| *Pericarditis* | 420.91 | I30.0 |  |  | ^1^ |  |  |  |
| *Cardiac Tamponade* | 423.3 | I31.4 |  |  | ^1^ |  |  |  |
| **Diabetes** |  |  |  |  |  |  |  |  |
| *Diabetes mellitus, Type I* |  |  | 249.00, 249.01, 249.10, 249.11, 249.20, 249.21, 249.30, 249.31, 249.40, 249.41, 249.50, 249.51, 249.60, 249.61, 249.70, 249.71, 249.80, 249.81, 249.90, 249.91, 250.00, 250.01, 250.02, 250.03, 250.10, 250.11, 250.12, 250.13, 250.20, 250.21, 250.22, 250.23, 250.30, 250.31, 250.32, 250.33, 250.40, 250.41, 250.42, 250.43, 250.50, 250.51, 250.52, 250.53, 250.60, 250.61, 250.62, 250.63, 250.70, 250.71, 250.72, 250.73, 250.80, 250.81, 250.82, 250.83, 250.90, 250.91, 250.92, 250.93, 357.2, 362.01, 362.02, 362.03, 362.04, 362.05, 362.06, 366.41 | E08.00, E08.01, E08.10, E08.11, E08.21, E08.22, E08.29, E08.311, E08.319, E08.321, E08.3211, E08.3212, E08.3213, E08.3219, E08.329, E08.3291, E08.3292, E08.3293, E08.3299, E08.331, E08.3311, E08.3312, E08.3313, E08.3319, E08.339, E08.3391, E08.3392, E08.3393, E08.3399, E08.341, E08.3411, E08.3412, E08.3413, E08.3419, E08.349, E08.3491, E08.3492, E08.3493, E08.3499, E08.351, E08.3511, E08.3512, E08.3513, E08.3519, E08.3521, E08.3522, E08.3523, E08.3529, E08.3531, E08.3532, E08.3533, E08.3539, E08.3541, E08.3542, E08.3543, E08.3549, E08.3551, E08.3552, E08.3553, E08.3559, E08.359, E08.3591, E08.3592, E08.3593, E08.3599, E08.36, E08.37X1, E08.37X2, E08.37X3, E08.37X9, E08.39, E08.40, E08.41, E08.42, E08.43, E08.44, E08.49, E08.51, E08.52, E08.59, E08.610, E08.618, E08.620, E08.621, E08.622, E08.628, E08.630, E08.638, E08.641, E08.649, E08.65, E08.69, E08.8, E08.9, E09.00, E09.01, E09.10, E09.11, E09.21, E09.22, E09.29, E09.311, E09.319, E09.321, E09.3211, E09.3212, E09.3213, E09.3219, E09.329, E09.3291, E09.3292, E09.3293, E09.3299, E09.331, E09.3311, E09.3312, E09.3313, E09.3319, E09.339, E09.3391, E09.3392, E09.3393, E09.3399, E09.341, E09.3411, E09.3412, E09.3413, E09.3419, E09.349, E09.3491, E09.3492, E09.3493, E09.3499, E09.351, E09.3511, E09.3512, E09.3513, E09.3519, E09.3521, E09.3522, E09.3523, E09.3529, E09.3531, E09.3532, E09.3533, E09.3539, E09.3541, E09.3542, E09.3543, E09.3549, E09.3551, E09.3552, E09.3553, E09.3559, E09.359, E09.3591, E09.3592, E09.3593, E09.3599, E09.36, E09.37X1, E09.37X2, E09.37X3, E09.37X9, E09.39, E09.40, E09.41, E09.42, E09.43, E09.44, E09.49, E09.51, E09.52, E09.59, E09.610, E09.618, E09.620, E09.621, E09.622, E09.628, E09.630, E09.638, E09.641, E09.649, E09.65, E09.69, E09.8, E09.9, E10.10, E10.11, E10.21, E10.22, E10.29, E10.311, E10.319, E10.321, E10.3211, E10.3212, E10.3213, E10.3219, E10.329, E10.3291, E10.3292, E10.3293, E10.3299, E10.331, E10.3311, E10.3312, E10.3313, E10.3319, E10.339, E10.3391, E10.3392, E10.3393, E10.3399, E10.341, E10.3411, E10.3412, E10.3413, E10.3419, E10.349, E10.3491, E10.3492, E10.3493, E10.3499, E10.351, E10.3511, E10.3512, E10.3513, E10.3519, E10.3521, E10.3522, E10.3523, E10.3529, E10.3531, E10.3532, E10.3533, E10.3539, E10.3541, E10.3542, E10.3543, E10.3549, E10.3551, E10.3552, E10.3553, E10.3559, E10.359, E10.3591, E10.3592, E10.3593, E10.3599, E10.36, E10.37X1, E10.37X2, E10.37X3, E10.37X9, E10.39, E10.40, E10.41, E10.42, E10.43, E10.44, E10.49, E10.51, E10.52, E10.59, E10.610, E10.618, E10.620, E10.621, E10.622, E10.628, E10.630, E10.638, E10.641, E10.649, E10.65, E10.69, E10.8, E10.9, E11.00, E11.01, E11.10, E11.11, E11.21, E11.22, E11.29, E11.311, E11.319, E11.321, E11.3211, E11.3212, E11.3213, E11.3219, E11.329, E11.3291, E11.3292, E11.3293, E11.3299, E11.331, E11.3311, E11.3312, E11.3313, E11.3319, E11.339, E11.3391, E11.3392, E11.3393, E11.3399, E11.341, E11.3411, E11.3412, E11.3413, E11.3419, E11.349, E11.3491, E11.3492, E11.3493, E11.3499, E11.351, E11.3511, E11.3512, E11.3513, E11.3519, E11.3521, E11.3522, E11.3523, E11.3529, E11.3531, E11.3532, E11.3533, E11.3539, E11.3541, E11.3542, E11.3543, E11.3549, E11.3551, E11.3552, E11.3553, E11.3559, E11.359, E11.3591, E11.3592, E11.3593, E11.3599, E11.36, E11.37X1, E11.37X2, E11.37X3, E11.37X9, E11.39, E11.40, E11.41, E11.42, E11.43, E11.44, E11.49, E11.51, E11.52, E11.59, E11.610, E11.618, E11.620, E11.621, E11.622, E11.628, E11.630, E11.638, E11.641, E11.649, E11.65, E11.69, E11.8, E11.9, E13.00, E13.01, E13.10, E13.11, E13.21, E13.22, E13.29, E13.311, E13.319, E13.321, E13.3211, E13.3212, E13.3213, E13.3219, E13.329, E13.3291, E13.3292, E13.3293, E13.3299, E13.331, E13.3311, E13.3312, E13.3313, E13.3319, E13.339, E13.3391, E13.3392, E13.3393, E13.3399, E13.341, E13.3411, E13.3412, E13.3413, E13.3419, E13.349, E13.3491, E13.3492, E13.3493, E13.3499, E13.351, E13.3511, E13.3512, E13.3513, E13.3519, E13.3521, E13.3522, E13.3523, E13.3529, E13.3531, E13.3532, E13.3533, E13.3539, E13.3541, E13.3542, E13.3543, E13.3549, E13.3551, E13.3552, E13.3553, E13.3559, E13.359, E13.3591, E13.3592, E13.3593, E13.3599, E13.36, E13.39, E13.40, E13.41, E13.42, E13.43, E13.44, E13.49, E13.51, E13.52, E13.59, E13.610, E13.618, E13.620, E13.621, E13.622, E13.628, E13.630, E13.638, E13.641, E13.649, E13.65, E13.69, E13.8, E13.9 | CCW | Diabetes |  |  |
| *Diabetes mellitus, Type II* |  |  |  |  |  |  |  |  |
| **Endocrine** |  |  |  |  |  |  |  |  |
| *Thyroid Disorder* |  |  | 244.0, 244.1, 244.2, 244.3, 244.8, 244.9 | E01.8, E02, E03.2, E03.3, E03.8, E03.9, E89.0 | CCW | Acquired Hypothyroidism |  |  |
|  | 245.9, 246.9, 242.90, 246.90 | E06.9, E07.9, E05.90 |  |  | ^1^ |  |  |  |
| *Pituitary Disorder* | 253.4, 256.4 | E23.6, E28.2 |  |  | ^1^ |  |  |  |
| **Gastrointestinal** |  |  |  |  |  |  |  |  |
| *Gallstones* | 574.50 | K80.50 |  |  | ^1^ |  |  |  |
| *Esophagitis* | 530.10 | K20.9 |  |  | ^1^ |  |  |  |
| *Gastric Ulcer* | 578.9, 530.20, 531.50, 533.40, 533.90, V12.71 | K92.2, K22.10, K25.5, K27.4, K27.9, Z87.11 |  |  | ^1^ |  |  |  |
| *Gastritis/Colitis* | 555.9, 558.9, 535.50, 562.11 | K50.90, K52.3, K52.89, K52.9, K29.70, K29.90, K57.32 |  |  | ^1^ |  |  |  |
| *Irritable Bowel Disease* | 564.1 | K58.1, K58.2, K58.8, K58.9 |  |  | ^1^ |  |  |  |
| *Stenosis of Rectum/Anus* | 569.2 | K62.4 |  |  | ^1^ |  |  |  |
| *Pancreatitis* | 577.1 | K86.1 |  |  | ^1^ |  |  |  |
| *History of Fistula* | - | - |  |  | ^1^ |  |  |  |
| *History of Digestive Congenital Disorder* | - | - |  |  |  |  |  |  |
| **HIV/AIDS** |  |  |  |  |  |  |  |  |
| *HIV Infection* |  |  | 042, 042.0, 042.1, 042.2, 042.9, 043, 043.1, 043.2, 043.3, 043.9, 044, 044.0, 044.9, 079.53, 795.71, V08; 795.71 requires a second qualifying claim that is not 795.71 (a screening code) | B20, B97.35, R75, Z21 R75 requires a second qualifying claim that is not R75 (a screening code) | CCW | Human Immunodeficiency Virus and/or Acquired Immunodeficiency Syndrome (HIV/AIDS) |  |  |
| **Hypertension** |  |  |  |  |  |  |  |  |
| *Hypertension* |  |  | 362.11, 401.0, 401.1, 401.9, 402.00, 402.01, 402.10, 402.11, 402.90, 402.91, 403.00, 403.01, 403.10, 403.11, 403.90, 403.91, 404.00, 404.01, 404.02, 404.03, 404.10, 404.11, 404.12, 404.13, 404.90, 404.91, 404.92, 404.93, 405.01, 405.09, 405.11, 405.19, 405.91, 405.99, 437.2 | H35.031, H35.032, H35.033, H35.039, I10, I11.0, I11.9, I12.0, I12.9, I13.0, I13.10, I13.11, I13.2, I15.0, I15.1, I15.2, I15.8, I15.9, I67.4, N26.2 | CCW | Hypertension |  |  |
| **Liver** |  |  |  |  |  |  |  |  |
| *Hepatitis B* |  |  | 070.3, 070.30, 070.31, 070.22, 070.23, 070.32, 070.33, V02.61 | B19.10, B19.11, B18.0, B18.1, Z22.51 | CCW | Hepatitis B (chronic or unspecified) |  |  |
| *Hepatitis C* |  |  | 070.7, 070.70, 070.71, 070.44, 070.54, V02.62 | B19.20, B19.21, B18.2, Z22.52 | CCW | Hepatitis C (chronic or unspecified) |  |  |
| *Non-alcoholic Cirrhosis* |  |  | 570, 571, 571.0, 571.1, 571.2, 571.3, 571.5, 571.6, 571.8, 571.9, 572, 572.0, 572.1, 572.2, 572.3, 572.4, 572.8, 573, 573.0, 573.4, 573.5, 573.8, 573.9, 576.1, 789.1, V42.7 ICD-9-CM Procedure Codes: 42.91, 44.91, 54.91, 96.06 | K70.0, K70.10, K70.11, K70.2, K70.30, K70.31, K70.40, K70.41, K70.9, K71.0, K71.11, K71.7, K71.8, K71.9, K72.00, K72.01, K72.10, K72.11, K72.90, K72.91, K74.0, K74.00, K74.01, K74.02, K74.1, K74.2, K74.3, K74.4, K74.5, K74.60, K74.69, K75.0, K75.1, K75.81, K75.89, K75.9, K76.0, K76.1, K76.2, K76.3, K76.5, K76.6, K76.7, K76.81, K76.82, K76.89, K76.9, K77, K80.30, K80.31, K80.32, K80.33, K80.34, K80.35, K80.36, K80.37, K83.0, R16.0, R16.2, Z48.23, Z94.4 ICD-10 Procedure Codes: 06L20ZZ, 06L23ZZ, 06L24ZZ, 06L30ZZ, 06L33ZZ, 06L34ZZ, 0DL57DZ, 0DL58DZ, 0D9S30Z, 0D9S3ZZ, 0D9S40Z, 0D9S4ZZ, 0D9T30Z, 0D9T3ZZ, 0D9T40Z, 0D9T4ZZ, 0D9V30Z, 0D9V3ZZ, 0D9V40Z, 0D9V4ZZ, 0D9W30Z, 0D9W3ZZ, 0D9W40Z, 0D9W4ZZ, 0W9F30Z, 0W9F3ZZ, 0W9F40Z, 0W9F4ZZ, 0W9G30Z, 0W9G3ZZ, 0W9G40Z, 0W9G4ZZ, 0W9J30Z, 0W9J3ZZ | CCW | Liver Disease, Cirrhosis and Other Liver Conditions (except Viral Hepatitis) |  |  |
| *Non-alcoholic Liver Disease* |  |  |  |  |  |  |  |  |
| **Mental Health** |  |  |  |  |  |  |  |  |
| *Mood Disorder* | 300.4, 300.9 | F34.1, F48.9, F99 |  |  | ^1^ |  |  |  |
|  |  |  | 309.0, 309.1, 309.22, 309.23, 309.24, 309.28, 309.29, 309.3, 309.4, 309.82, 309.83, 309.89, 309.9 | F43.20, F43.21, F43.22, F43.23, F43.24, F43.25, F43.29, F43.8, F43.9 | HCUP^3^ | Adjustment disorders |  |  |
|  |  |  | 295.00, 295.01, 295.02, 295.03, 295.04, 295.05, 295.10, 295.11, 295.12, 295.13, 295.14, 295.15, 295.20, 295.21, 295.22, 295.23, 295.24, 295.25, 295.30, 295.31, 295.32, 295.33, 295.34, 295.35, 295.40, 295.41, 295.42, 295.43, 295.44, 295.45, 295.50, 295.51, 295.52, 295.53, 295.54, 295.55, 295.60, 295.61, 295.62, 295.63, 295.64, 295.65, 295.70, 295.71, 295.72, 295.73, 295.74, 295.75, 295.80, 295.81, 295.82, 295.83, 295.84, 295.85, 295.90, 295.91, 295.92, 295.93, 295.94, 295.95 | F20.0, F20.1, F20.2, F20.3, F20.5, F20.81, F20.89, F20.9, F25.0, F25.1, F25.8, F25.9 | CCW | Schizophrenia |  |  |
|  |  |  | 296.00, 296.01, 296.02, 296.03, 296.04, 296.05, 296.06, 296.10, 296.11, 296.12, 296.13, 296.14, 296.15, 296.16, 296.40, 296.41, 296.42, 296.43, 296.44, 296.45, 296.46, 296.50, 296.51, 296.52, 296.53, 296.54, 296.55, 296.56, 296.60, 296.61, 296.62, 296.63, 296.64, 296.65, 296.66, 296.7, 296.80, 296.81, 296.82, 296.89, 296.90, 296.99 | F30.10, F30.11, F30.12, F30.13, F30.2, F30.3, F30.4, F30.8, F30.9, F31.0, F31.10, F31.11, F31.12, F31.13, F31.2, F31.30, F31.31, F31.32, F31.4, F31.5, F31.60, F31.61, F31.62, F31.63, F31.64, F31.70, F31.71, F31.72, F31.73, F31.74, F31.75, F31.76, F31.77, F31.78, F31.81, F31.89, F31.9, F33.8, F34.81, F34.89, F34.9, F39 | CCW | Bipolar Disorder |  |  |
|  | 296.90, 300.01, 309.24 | F39, F41.0, F43.22 |  |  | ^1^ |  |  |  |
| *Depressive Disorder* |  |  | 296.20, 296.21, 296.22, 296.23, 296.24, 296.25, 296.26, 296.30, 296.31, 296.32, 296.33, 296.34, 296.35, 296.36, 300.4, 311 | F32.0, F32.1, F32.2, F32.3, F32.4, F32.5, F32.89, F32.9, F32.A, F33.0, F33.1, F33.2, F33.3, F33.40, F33.41, F33.42, F33.8, F33.9, F34.1 | CCW | Depressive Disorders |  |  |
| *Anxiety Disorder* |  |  | 293.84, 300.00, 300.01, 300.02, 300.09, 300.10, 300.20, 300.21, 300.22, 300.23, 300.29, 300.3, 300.5, 300.89, 300.9, 308.0, 308.1, 308.2, 308.3, 308.4, 308.9, ~~309.81~~, 313.0, 313.1, 313.21, 313.22, 313.3, 313.82, 313.83 | F06.4, F40.00, F40.01, F40.02, F40.10, F40.11, F40.210, F40.218, F40.220, F40.228, F40.230, F40.231, F40.232, F40.233, F40.240, F40.241, F40.242, F40.243, F40.248, F40.290, F40.291, F40.298, F40.8, F40.9, F41.0, F41.1, F41.3, F41.8, F41.9, F42, F42.2, F42.3, F42.4, F42.8, F42.9, F43.0, ~~F43.10, F43.11, F43.12~~, F44.9, F45.8, F48.8, F48.9, F93.8, F99, R45.2, R45.5, R45.6, R45.7 | CCW | Anxiety Disorders |  |  |
| *Attention Deficit Disorder* |  |  | 312.00, 312.01, 312.02, 312.03, 312.10, 312.11, 312.12, 312.13, 312.20, 312.21, 312.22, 312.23, 312.30, 312.31, 312.32, 312.33, 312.34, 312.35, 312.39, 312.4, 312.81, 312.82, 312.89, 312.9, 314.00, 314.01, 314.1, 314.2, 314.8, 314.9 | F63.0, F63.1, F63.2, F63.3, F63.81, F63.89, F63.9, F90.0, F90.1, F90.2, F90.8, F90.9, F91.0, F91.1, F91.2, F91.3, F91.8, F91.9 | CCW | ADHD, Conduct Disorders, and Hyperkinetic Syndrome |  |  |
| *Mental Disability* | - | - |  |  |  |  |  |  |
| **Neurological** |  |  |  |  | ^1^ |  |  |  |
| *Cerebrovascular/Stroke* |  |  | 430, 431, 433.01, 433.11, 433.21, 433.31, 433.81, 433.91, 434.00, 434.01, 434.10, 434.11, 434.90, 434.91, 435.0, 435.1, 435.3, 435.8, 435.9, 436, 997.02; EXCLUSION: If any of the qualifying claims have: 800 <= DX Code <= 804.99, 850 <= DX Code <= 854.19 in any DX position OR DX V57xx as the principal DX Code, then EXCLUDE. | G45.0, G45.1, G45.2, G45.8, G45.9, G46.0, G46.1, G46.2, G46.3, G46.4, G46.5, G46.6, G46.7, G46.8, G97.31, G97.32, I60.00, I60.01, I60.02, I60.10, I60.11, I60.12, I60.20, I60.21, I60.22, I60.30, I60.31, I60.32, I60.4, I60.50, I60.51, I60.52, I60.6, I60.7, I60.8, I60.9, I61.0, I61.1, I61.2, I61.3, I61.4, I61.5, I61.6, I61.8, I61.9, I63.00, I63.011, I63.012, I63.013, I63.019, I63.02, I63.031, I63.032, I63.039, I63.09, I63.10, I63.111, I63.112, I63.113, I63.119, I63.12, I63.131, I63.132, I63.133, I63.139, I63.19, I63.20, I63.211, I63.212, I63.213, I63.219, I63.22, I63.231, I63.232, I63.233, I63.239, I63.29, I63.30, I63.311, I63.312, I63.313, I63.319, I63.321, I63.322, I63.323, I63.329, I63.331, I63.332, I63.333, I63.339, I63.341, I63.342, I63.343, I63.349, I63.39, I63.40, I63.411, I63.412, I63.413, I63.419, I63.421, I63.422, I63.423, I63.429, I63.431, I63.432, I63.433, I63.439, I63.441, I63.442, I63.443, I63.449, I63.49, I63.50, I63.511, I63.512, I63.513, I63.519, I63.521, I63.522, I63.523, I63.529, I63.531, I63.532, I63.533, I63.539, I63.541, I63.542, I63.543, I63.549, I63.59, I63.6, I63.8, I63.81, I63.89, I63.9, I66.01, I66.02, I66.03, I66.09, I66.11, I66.12, I66.13, I66.19, I66.21, I66.22, I66.23, I66.29, I66.3, I66.8, I66.9, I67.841, I67.848, I67.89, I97.810, I97.811, I97.820, I97.821; EXCLUSION: If any of the qualifying claims have any of the following codes in any DX position then EXCLUDE: S01.90XA, S02.0XXA, S02.0XXB, S02.101A, S02.101B, S02.102A, S02.102B, S02.109A, S02.109B, S02.10XA, S02.10XB, S02.110A, S02.110B, S02.111A, S02.111B, S02.112A, S02.112B, S02.113A, S02.113B, S02.118A, S02.118B, S02.119A, S02.119B, S02.11GA, S02.11GB, S02.11HA, S02.11HB, S02.121A, S02.121B, S02.121D, S02.121G, S02.121K, S02.121S, S02.122A, S02.122B, S02.122D, S02.122G, S02.122K, S02.122S, S02.129A, S02.129B, S02.129D, S02.129G, S02.129K, S02.129S, S02.19XA, S02.19XB, S02.2XXA, S02.2XXB, S02.30XA, S02.30XB, S02.31XA, S02.31XB, S02.32XA, S02.32XB, S02.3XXA, S02.3XXB, S02.400A, S02.400B, S02.401A, S02.401B, S02.402A, S02.402B, S02.40AA, S02.40AB, S02.40BA, S02.40BB, S02.40CA, S02.40CB, S02.40DA, S02.40DB, S02.40EA, S02.40EB, S02.40FA, S02.40FB, S02.411A, S02.411B, S02.412A, S02.412B, S02.413A, S02.413B, S02.42XA, S02.42XB, S02.600A, S02.600B, S02.601A, S02.601B, S02.602A, S02.602B, S02.609A, S02.609B, S02.610A, S02.610B, S02.611A, S02.611B, S02.612A, S02.612B, S02.61XA, S02.61XB, S02.620A, S02.620B, S02.621A, S02.621B, S02.622A, S02.622B, S02.62XA, S02.62XB, S02.630A, S02.630B, S02.631A, S02.631B, S02.632A, S02.632B, S02.63XA, S02.63XB, S02.640A, S02.640B, S02.641A, S02.641B, S02.642A, S02.642B, S02.64XA, S02.64XB, S02.650A, S02.650B, S02.651A, S02.651B, S02.652A, S02.652B, S02.65XA, S02.65XB, S02.66XA, S02.66XB, S02.670A, S02.670B, S02.671A, S02.671B, S02.672A, S02.672B, S02.67XA, S02.67XB, S02.69XA, S02.69XB, S02.80XA, S02.80XB, S02.81XA, S02.81XB, S02.82XA, S02.82XB, S02.831A, S02.831B, S02.831D, S02.831G, S02.831K, S02.831S, S02.832A, S02.832B, S02.832D, S02.832G, S02.832K, S02.832S, S02.839A, S02.839B, S02.839D, S02.839G, S02.839K, S02.839S, S02.841A, S02.841B, S02.841D, S02.841G, S02.841K, S02.841S, S02.842A, S02.842B, S02.842D, S02.842G, S02.842K, S02.842S, S02.849A, S02.849B, S02.849D, S02.849G, S02.849K, S02.849S, S02.85XA, S02.85XB, S02.85XD, S02.85XG, S02.85XK, S02.85XS, S02.8XXA, S02.8XXB, S02.91XA, S02.91XB, S02.92XA, S02.92XB, S06.0X0A, S06.0X1A, S06.0X2A, S06.0X3A, S06.0X4A, S06.0X5A, S06.0X6A, S06.0X7A, S06.0X8A, S06.0X9A, S06.1X0A, S06.1X1A, S06.1X2A, S06.1X3A, S06.1X4A, S06.1X5A, S06.1X6A, S06.1X7A, S06.1X8A, S06.1X9A, S06.2X0A, S06.2X1A, S06.2X2A, S06.2X3A, S06.2X4A, S06.2X5A, S06.2X6A, S06.2X7A, S06.2X8A, S06.2X9A, S06.300A, S06.301A, S06.302A, S06.303A, S06.304A, S06.305A, S06.306A, S06.307A, S06.308A, S06.309A, S06.310A, S06.311A, S06.312A, S06.313A, S06.314A, S06.315A, S06.316A, S06.317A, S06.318A, S06.319A, S06.320A, S06.321A, S06.322A, S06.323A, S06.324A, S06.325A, S06.326A, S06.327A, S06.328A, S06.329A, S06.330A, S06.331A, S06.332A, S06.333A, S06.334A, S06.335A, S06.336A, S06.337A, S06.338A, S06.339A, S06.340A, S06.341A, S06.342A, S06.343A, S06.344A, S06.345A, S06.346A, S06.347A, S06.348A, S06.349A, S06.350A, S06.351A, S06.352A, S06.353A, S06.354A, S06.355A, S06.356A, S06.357A, S06.358A, S06.359A, S06.360A, S06.361A, S06.362A, S06.363A, S06.364A, S06.365A, S06.366A, S06.367A, S06.368A, S06.369A, S06.370A, S06.371A, S06.372A, S06.373A, S06.374A, S06.375A, S06.376A, S06.377A, S06.378A, S06.379A, S06.380A, S06.381A, S06.382A, S06.383A, S06.384A, S06.385A, S06.386A, S06.387A, S06.388A, S06.389A, S06.4X0A, S06.4X1A, S06.4X2A, S06.4X3A, S06.4X4A, S06.4X5A, S06.4X6A, S06.4X7A, S06.4X8A, S06.4X9A, S06.5X0A, S06.5X1A, S06.5X2A, S06.5X3A, S06.5X4A, S06.5X5A, S06.5X6A, S06.5X7A, S06.5X8A, S06.5X9A, S06.6X0A, S06.6X1A, S06.6X2A, S06.6X3A, S06.6X4A, S06.6X5A, S06.6X6A, S06.6X7A, S06.6X8A, S06.6X9A, S06.810A, S06.811A, S06.812A, S06.813A, S06.814A, S06.815A, S06.816A, S06.817A, S06.818A, S06.819A, S06.820A, S06.821A, S06.822A, S06.823A, S06.824A, S06.825A, S06.826A, S06.827A, S06.828A, S06.829A, S06.890A, S06.891A, S06.892A, S06.893A, S06.894A, S06.895A, S06.896A, S06.897A, S06.898A, S06.899A, S06.9X0A, S06.9X1A, S06.9X2A, S06.9X3A, S06.9X4A, S06.9X5A, S06.9X6A, S06.9X7A, S06.9X8A, S06.9X9A, OR Z51.89 as the principal DX Code then EXCLUDE. | CCW | Stroke/Transient Ischemic Attack |  |  |
|  |  |  |  |  |  |  |  | |
| *Chronic Pain* |  |  | 338.2, 338.21, 338.22, 338.28, 338.29, 338.3, 338.4, 780.7, 780.71, 729.1, 729.2 | G89.21, G89.22, G89.28, G89.29, G89.3, G89.4, M54.10, M54.11, M54.12, M54.13, M54.14, M54.15, M54.16, M54.17, M54.18, M60.80, M60.811, M60.812, M60.819, M60.821, M60.822, M60.829, M60.831, M60.832, M60.839, M60.841, M60.842, M60.849, M60.851, M60.852, M60.859, M60.861, M60.862, M60.869, M60.871, M60.872, M60.879, M60.88, M60.89, M60.9, M79.1, M79.10, M79.11, M79.12, M79.18, M79.2, M79.7, R53.82 | CCW | Fibromyalgia, Chronic Pain and Fatigue |  | |
| *Headaches* |  |  | 339, 339.0, 339.00, 339.01, 339.02, 339.03, 339.04, 339.05, 339.09, 339.1, 339.10, 339.11, 339.12, 339.2, 339.20, 339.21, 339.22, 339.3, 339.4, 339.41, 339.42, 339.43, 339.44, 339.8, 339.81, 339.82, 339.83, 339.84, 339.85, 339.89, 346, 346.0, 346.00, 346.01, 346.02, 346.03, 346.1, 346.10, 346.11, 346.12, 346.13, 346.2, 346.20, 346.21, 346.22, 346.23, 346.3, 346.30, 346.31, 346.32, 346.33, 346.4, 346.40, 346.41, 346.42, 346.43, 346.5, 346.50, 346.51, 346.52, 346.53, 346.6, 346.60, 346.61, 346.62, 346.63, 346.7, 346.70, 346.71, 346.72, 346.73, 346.8, 346.80, 346.81, 346.82, 346.83, 346.9, 346.90, 346.91, 346.92, 346.93 | G43.001, G43.009, G43.011, G43.019, G43.101, G43.109, G43.111, G43.119, G43.401, G43.409, G43.411, G43.419, G43.501, G43.509, G43.511, G43.519, G43.601, G43.609, G43.611, G43.619, G43.701, G43.709, G43.711, G43.719, G43.A0, G43.B0, G43.C0, G43.D0, G43.A1, G43.B1, G43.C1, G43.D1, G43.801, G43.809, G43.811, G43.819, G43.821, G43.829, G43.831, G43.839, G43.901, G43.909, G43.911, G43.919, G44.001, G44.009, G44.011, G44.019, G44.021, G44.029, G44.031, G44.039, G44.041, G44.049, G44.051, G44.059, G44.091, G44.099, G44.1, G44.201, G44.209, G44.211, G44.219, G44.221, G44.229, G44.301, G44.309, G44.311, G44.319, G44.321, G44.329, G44.40, G44.41, G44.51, G44.52, G44.53, G44.59, G44.81, G44.82, G44.83, G44.84, G44.85, G44.86, G44.89 | CCW | Migraine and Other Chronic Headache |  | |
| *Multiple Sclerosis* |  |  | 340, 341, 341.0, 341.2, 341.20, 341.21, 341.22, 341.8, 341.9 | G35, G36.0, G36.1, G36.8, G36.9, G37.1, G37.2, G37.3, G37.4, G37.8, G37.9 | CCW | Multiple Sclerosis and Transverse Myelitis |  | |
| *Cerebral Palsy* |  |  | 333.71, 343, 343.0, 343.1, 343.2, 343.3, 343.4, 343.8, 343.9 | G80.0, G80.1, G80.2, G80.3, G80.4, G80.8, G80.9 | CCW | Cerebral Palsy |  | |
| *Epilepsy* |  |  | 345, 345.0, 345.00, 345.01, 345.1, 345.10, 345.11, 345.2, 345.3, 345.4, 345.40, 345.41, 345.5, 345.50, 345.51, 345.6, 345.60, 345.61, 345.7, 345.70, 345.71, 345.8, 345.80, 345.81, 345.9, 345.90, 345.91 | G40.001, G40.009, G40.011, G40.019, G40.101, G40.109, G40.111, G40.119, G40.201, G40.209, G40.211, G40.219, G40.301, G40.309, G40.311, G40.319, G40.401, G40.409, G40.411, G40.419, G40.42, G40.501, G40.509, G40.801, G40.802, G40.803, G40.804, G40.811, G40.812, G40.813, G40.814, G40.821, G40.822, G40.823, G40.824, G40.833, G40.834, G40.89, G40.901, G40.909, G40.911, G40.919, G40.A01, G40.A09, G40.A11, G40.A19, G40.B01, G40.B09, G40.B11, G40.B19 | CCW | Epilepsy |  | |
| *Neuropathy* | 356.9, 782.0 | G60.9, R20.0, R20.1, R20.2, R20.3, R20.8, R20.9 |  |  | ^1^ |  |  | |
| *Eye Issues* | 368.2, 362.17, 377.30 | H53.2, H35.09, H46.9 |  |  | ^1^ |  |  | |
| *Congenital Neurological Deficit* |  |  | 740.0, 740.1, 740.2, 741, 741.0, 741.00, 741.01, 741.02, 741.03, 741.9, 741.90, 741.91, 741.92, 741.93, 742.0, 742.1, 742.2, 742.3, 742.4, 742.5, 742.51, 742.53, 742.59, 742.8, 742.9 | G90.1, Q00.0, Q00.1, Q00.2, Q01.0, Q01.1, Q01.2, Q01.8, Q01.9, Q02, Q03.0, Q03.1, Q03.8, Q03.9, Q04.0, Q04.1, Q04.2, Q04.3, Q04.4, Q04.5, Q04.6, Q04.8, Q04.9, Q05.0, Q05.1, Q05.2, Q05.3, Q05.4, Q05.5, Q05.6, Q05.7, Q05.8, Q05.9, Q06.0, Q06.1, Q06.2, Q06.3, Q06.4, Q06.8, Q06.9, Q07.00, Q07.01, Q07.02, Q07.03, Q07.8, Q07.9 | CCW | Spina Bifida and Other Congenital Anomalies of the Nervous System |  | |
| *Syncope* | 780.2, 780.4 | R55, R42 |  |  | ^1^ |  |  | |
| *Compression of Brain* | 348.4 | G93.5 |  |  | ^1^ |  |  | |
| *Cranial Nerve Issue* | 351.0, 781.94 | G51.0, R29.810 |  |  | ^1^ |  |  | |
| *Extremity Issue* | 354.0, 723.4, 719.7 | G56.00, M54.12, M54.13, R26.2 |  |  | ^1^ |  |  | |
| *Developmental Delay* |  |  | 315.5, 315.8, 315.9 | F81.9, F82, F88, F89 | CCW | Other Developmental Delays |  | |
| *Altered Mental State* | 780.97 | R41.82 |  |  | ^1^ |  |  | |
| **Obesity/Overweight** |  |  |  |  |  |  |  | |
| *Overweight/Obese* |  |  | 278.0, 278.00, 278.01, 278.03, V85.3, V85.30, V85.31, V85.32, V85.33, V85.34, V85.35, V85.36, V85.37, V85.38, V85.39, V85.4, V85.41, V85.42, V85.43, V85.44, V85.45 | E66.01, E66.09, E66.1, E66.2, E66.8, E66.9, Z68.30, Z68.31, Z68.32, Z68.33, Z68.34, Z68.35, Z68.36, Z68.37, Z68.38, Z68.39, Z68.41, Z68.42, Z68.43, Z68.44, Z68.45 | CCW | Obesity |  | |
| **Renal** |  |  |  |  |  |  |  | |
| *Renal Disease* |  |  | 016.00, 016.01, 016.02, 016.03, 016.04, 016.05, 016.06, 095.4, 189.0, 189.9, 223.0, 236.91, 249.40, 249.41, 250.40, 250.41, 250.42, 250.43, 271.4, 274.10, 283.11, 403.01, 403.11, 403.91, 404.02, 404.03, 404.12, 404.13, 404.92, 404.93, 440.1, 442.1, 572.4, 580.0, 580.4, 580.81, 580.89, 580.9, 581.0, 581.1, 581.2, 581.3, 581.81, 581.89, 581.9, 582.0, 582.1, 582.2, 582.4, 582.81, 582.89, 582.9, 583.0, 583.1, 583.2, 583.4, 583.6, 583.7, 583.81, 583.89, 583.9, 584.5, 584.6, 584.7, 584.8, 584.9, 585.1, 585.2, 585.3, 585.4, 585.5, 585.6, 585.9, 586, 587, 588.0, 588.1, 588.81, 588.89, 588.9, 591, 753.12, 753.13, 753.14, 753.15, 753.16, 753.17, 753.19, 753.20, 753.21, 753.22, 753.23, 753.29, 794.4 | A18.11, A52.75, B52.0, C64.1, C64.2, C64.9, C68.9, D30.00, D30.01, D30.02, D41.00, D41.01, D41.02, D41.10, D41.11, D41.12, D41.20, D41.21, D41.22, D59.3, E08.21, E08.22, E08.29, E08.65, E09.21, E09.22, E09.29, E10.21, E10.22, E10.29, E10.65, E11.21, E11.22, E11.29, E11.65, E13.21, E13.22, E13.29, E74.8, I12.0, I12.9, I13.0, I13.10, I13.11, I13.2, I70.1, I72.2, K76.7, M10.30, M10.311, M10.312, M10.319, M10.321, M10.322, M10.329, M10.331, M10.332, M10.339, M10.341, M10.342, M10.349, M10.351, M10.352, M10.359, M10.361, M10.362, M10.369, M10.371, M10.372, M10.379, M10.38, M10.39, M32.14, M32.15, M35.04, N00.0, N00.1, N00.2, N00.3, N00.4, N00.5, N00.6, N00.7, N00.8, N00.9, N00.A, N01.0, N01.1, N01.2, N01.3, N01.4, N01.5, N01.6, N01.7, N01.8, N01.9, N01.A, N02.0, N02.1, N02.2, N02.3, N02.4, N02.5, N02.6, N02.7, N02.8, N02.9, N02.A, N03.0, N03.1, N03.2, N03.3, N03.4, N03.5, N03.6, N03.7, N03.8, N03.9, N03.A, N04.0, N04.1, N04.2, N04.3, N04.4, N04.5, N04.6, N04.7, N04.8, N04.9, N04.A, N05.0, N05.1, N05.2, N05.3, N05.4, N05.5, N05.6, N05.7, N05.8, N05.9, N05.A, N06.0, N06.1, N06.2, N06.3, N06.4, N06.5, N06.6, N06.7, N06.8, N06.9, N06.A, N07.0, N07.1, N07.2, N07.3, N07.4, N07.5, N07.6, N07.7, N07.8, N07.9, N07.A, N08, N13.1, N13.2, N13.30, N13.39, N14.0, N14.1, N14.2, N14.3, N14.4, N15.0, N15.8, N15.9, N16, N17.0, N17.1, N17.2, N17.8, N17.9, N18.1, N18.2, N18.3, N18.30, N18.31, N18.32, N18.4, N18.5, N18.6, N18.9, N19, N25.0, N25.1, N25.81, N25.89, N25.9, N26.1, N26.9, Q61.02, Q61.11, Q61.19, Q61.2, Q61.3, Q61.4, Q61.5, Q61.8, Q62.0, Q62.2, Q62.10, Q62.11, Q62.12, Q62.31, Q62.32, Q62.39, R94.4 | CCW | Chronic Kidney Disease |  | |
| *Kidney Transplant* |  |  |  |  |  |  |  | |
| *Solitary Kidney* |  |  |  |  |  |  |  | |
| **Ashma/Respiratory** |  |  |  |  |  |  |  | |
| *Asthma* |  |  | 493.00, 493.01, 493.02, 493.10, 493.11, 493.12, 493.20, 493.21, 493.22, 493.81, 493.82, 493.90, 493.91, 493.92 | J45.20, J45.21, J45.22, J45.30, J45.31, J45.32, J45.40, J45.41, J45.42, J45.50, J45.51, J45.52, J45.901, J45.902, J45.909, J45.990, J45.991, J45.998, J82.83 | CCW | Asthma |  | |
| *COPD* |  |  | 490, 491.0, 491.1, 491.20, 491.21, 491.22, 491.8, 491.9, 492.0, 492.8, 494.0, 494.1, 496 | J40, J41.0, J41.1, J41.8, J42, J43.0, J43.1, J43.2, J43.8, J43.9, J44.0, J44.1, J44.9, J47.0, J47.1, J47.9 | CCW | Chronic Obstructive Pulmonary Disease and Bronchiectasis |  | |
| **Rheumatologic/**  **Autoimmune** |  |  |  |  |  |  |  | |
| *Rheumatoid Disease* | 701.0, 714.30, 715.90 | L90.0, L94.0, L94.3, M08.00, M15.9, M19.90 |  |  | ^1^ |  |  | |
| **Hematologic** |  |  |  |  |  |  |  | |
| *Hereditary Spherocytosi* | 282.0 | D58.0 |  |  | ^1^ |  |  | |
| *Sickle-cell Disease* |  |  | 282.41, 282.42, 282.60, 282.61, 282.62, 282.63, 282.64, 282.68, 282.69 | D57.00, D57.01, D57.02, D57.03, D57.09, D57.1, D57.20, D57.211, D57.212, D57.213, D57.218, D57.219, D57.40, D57.411, D57.412, D57.413, D57.418, D57.419, D57.42, D57.431, D57.432, D57.433, D57.438, D57.439, D57.44, D57.451, D57.452, D57.453, D57.458, D57.459, D57.80, D57.811, D57.812, D57.813, D57.818, D57.819 | CCW | Sickle Cell Disease |  | |
| *von Willebrand’s Disease* | 286.4 | D68.0 |  |  | ^1^ |  |  | |
| *Immune Thrombocyotpenic Purpura* | 287.31 | D69.3 |  |  | ^1^ |  |  | |
|  |  |  |  |  |  |  |  | |
| 1. Wu XC, Prasad PK, Landry I, Harlan LC, Parsons HM, Lynch CF, Smith AW, Hamilton AS, Keegan TH; AYA HOPE Study Collaborative Group. Impact of the AYA HOPE Comorbidity Index on Assessing Health Care Service Needs and Health Status among Adolescents and Young Adults with Cancer. Cancer Epidemiol Biomarkers Prev. 2015 Dec;24(12):1844-9. doi: 10.1158/1055-9965.EPI-15-0401. Epub 2015 Sep 29. PMID: 26420768; PMCID: PMC4670595. | | | | | | |  | |
