# Supplementary material for: Autism, Obesity, and PTSD Among Adolescents and Young Adults: An Analysis of National Medicaid Claims Data
Source: J Autism Dev Disord. Author manuscript; Available in PMC 2025 Sep 15. (PMC12434421; doi:10.1007/s10803-025-06881-1)
Supplement: Supplementary Material 3 [file NIHMS2101657-supplement-Supplementary_Material_3.docx]

**SM3. Demographics for Medicaid enrollees age 15-30 by ASD/ID conditions in 2008-2019 by Sex**

|  | **Autistic** | | | | **Non-Autistic** | | | |
| --- | --- | --- | --- | --- | --- | --- | --- | --- |
|  | **Male** | | **Female** | | **Male** | | **Female** | |
|  | N = 474940 | | N = 152646 | | N = 445278 | | N = 777883 | |
|  | N | % | N | % | N | % | N | % |
| **PTSD** | 24,100 | 5.07 | 15,868 | 10.40 | 6,706 | 1.51 | 20,566 | 2.64 |
| **Obesity** | 56,489 | 11.89 | 26,417 | 17.31 | 17,518 | 3.93 | 66,193 | 8.51 |
| **HOPE (binary)** |  |  |  |  |  |  |  |  |
| Less Severe | 412,145 | 86.78 | 122,950 | 80.55 | 430,433 | 96.67 | 730,149 | 93.86 |
| More Severe | 62,795 | 13.22 | 29,696 | 19.45 | 14,845 | 3.33 | 47,734 | 6.14 |
