# Supplementary material for: Autism, Obesity, and PTSD Among Adolescents and Young Adults: An Analysis of National Medicaid Claims Data
Source: J Autism Dev Disord. Author manuscript; Available in PMC 2025 Sep 15. (PMC12434421; doi:10.1007/s10803-025-06881-1)
Supplement: Supplementary Material 4 [file NIHMS2101657-supplement-Supplementary_Material_4.docx]

**SM4. Prevalence and Odds of Obesity and HOPE more severe condition among Medicaid enrollees (age 15-30) among autistic and non-autistic beneficiaries (2008-2019) by sex**

|  |  | **Autistic** | | | | | | | | **Non-Autistic** | |
| --- | --- | --- | --- | --- | --- | --- | --- | --- | --- | --- | --- |
|  |  | **N** | **%** | **uOR^1^** | **95% CI** | | **aOR^2^** | **95% CI** | | **N** | **%** |
| **Obesity** | **Male** | 56,489 | 11.89 | 3.296 | 3.239 | 3.354 | 2.674 | 2.616 | 2.733 | 17,518 | 3.93 |
|  | **Female** | 26,417 | 17.31 | 2.250 | 2.216 | 2.285 | 1.755 | 1.717 | 1.794 | 66,193 | 8.51 |
| **HOPE^3^** | **Male** | 62,795 | 13.22 | 4.418 | 4.337 | 4.500 | 2.408 | 2.355 | 2.463 | 14,845 | 3.33 |
|  | **Female** | 29,696 | 19.45 | 3.694 | 3.637 | 3.753 | 1.875 | 1.832 | 1.918 | 47,734 | 6.14 |

| ^1^ Unadjusted OR comparing autistic versus non-autistic beneficiaries. |
| --- |
| ^2^ Logistic regression adjusted for age group, sex, race/ethnicity, Medicaid eligibility group, enrolled month group, and state. |
| ^3^ Odds comparing more severe condition to less severe condition. |
